# Diffusion MRI: what water tells us about the brain

**DOI:** 10.1002/emmm.201404055

**Published:** 2014-04-04

**Authors:** Denis Le Bihan

**Affiliations:** NeuroSpin, CEA Saclay-CenterGif-sur-Yvette, France

## Abstract

Diffusion MRI has been used worldwide to produce images of brain tissue structure and connectivity, in the normal and diseased brain. Diffusion MRI has revolutionized the management of acute brain ischemia (stroke), saving life of many patients and sparing them significant disabilities. In addition to stroke, diffusion MRI is now widely used for the detection of cancers and metastases (breast, prostate, liver). Another major field of application of diffusion MRI regards the wiring of the brain. Diffusion MRI is now used to map the circuitry of the human brain with incredible accuracy, opening up new lines of inquiry for human neuroscience and for the understanding of brain illnesses or mental disorders. Here, as a pioneer of the field, I provide a personal account on the historical development of these concepts over the last 30 years.

Among the sensational 1905 Albert Einstein papers, there is one that unexpectedly gave birth to a powerful method to explore the brain. Einstein explained molecular diffusion on the basis of the random translational motion of molecules, which results from their thermal energy (Einstein, 1905). Moving fast forward, in the mid 1980s, I was able to show that water diffusion could be imaged in the human brain through magnetic resonance imaging (MRI). This move was triggered by the idea that water diffusion could provide unique information on the functional architecture of tissues, since during their random displacements water molecules probe tissue structure at a microscopic scale (Fig 1). What I did not expect was that this pioneering work would end up some day interesting the readers of a major molecular medicine journal. Sure, this story is about an apparently simple molecule, water. However, although water is an essential molecule for life, its importance in biology has perhaps been often overlooked, if not forgotten. Water diffusion MRI has proven to be extraordinarily popular. Its main clinical domain of application (Fig 2) has been neurological disease, especially in the management of patients with acute brain ischemia. With its unmatched sensitivity, water diffusion MRI provides patients with the opportunity to receive suitable thrombolytic treatment at a stage when brain tissue might still be salvageable, thus avoiding them terrible consequences. On the other hand, water diffusion turned out to be anisotropic in brain white matter, because axon membranes limit molecular movement perpendicularly to the axonal fibers. This feature can be exploited to produce stunning maps of the organization in space of white matter bundles and brain connections in just a few minutes, as well as to provide information on white matter integrity. Diffusion MRI has been also used in a full-body setting for the detection and treatment monitoring of cancer lesions and metastases (liver, breast, prostate), because water diffusion slows down in malignant tissues in relation to the cell proliferation. The versatility and the potential of diffusion MRI, both for research and clinical applications, have been reviewed elsewhere (Le Bihan, 2003; Le Bihan & Johansen-Berg, 2012). Here, I will provide a more personal account on the historical development of these concepts and how they inspired my research in the last 30 years.

**Figure 1:**
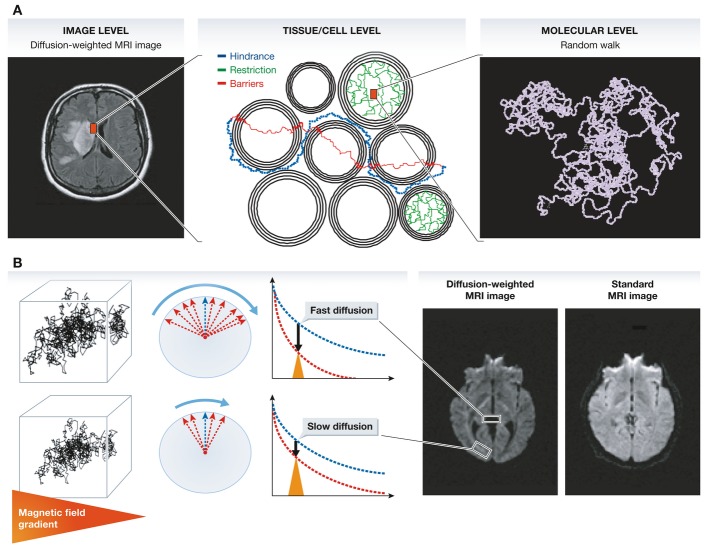
(A) Contrast and signal levels in the diffusion-weighted image (left) reflect water diffusion behavior (random walk) (right). Diffusion behavior is modulated by tissue structure at the cellular level (middle): For instance, diffusion can be restricted within cells, water may escape when cell membranes are permeable and might then experience a tortuous pathway in the extracellular space (hindrance). (B) In the presence of a magnetic field gradient (variation of the magnetic field along one spatial direction), magnetized water molecule hydrogen atoms are dephased. The amount of dephasing is directly related to the diffusion distance (a few micrometers) covered by water molecules during measurement (a few tens of milliseconds). Given the great many water molecules experiencing individual random walk displacements, the overall effect of this dephasing is an interference, which reduces MRI signal amplitudes. In areas with fast water diffusion (e.g. within ventricules), the signal is deeply reduced, while in areas of slow water diffusion (e.g. white matter bundles), the signal is only slightly reduced. This differential effect results in a contrast in the diffusion-weighted MRI images, which is not visible in standard MRI images.

**Figure 2:**
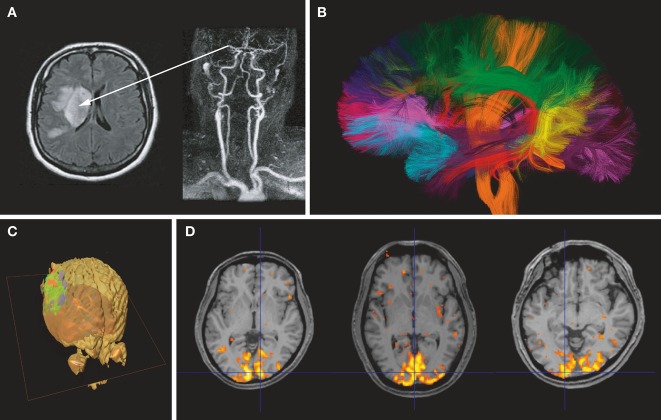
(A) *Acute stroke*. The angiogram (right) shows an occlusion of the right middle cerebral artery. The diffusion-weighted image (left) clearly shows a bright signal corresponding to a drop in water diffusion resulting from cell swelling (cytotoxic edema) in the tissue undergoing acute ischemia. (B) *Brain connectivity*. Water diffusion in white matter fibers is anisotropic, faster in the direction of the fibers. By measuring water diffusion in many directions, the orientations of the whiter matter bundles can be retrieved at each brain location. Algorithms are then used to identify bundles, which are represented with arbitrary colors (courtesy CONNECT/NeuroSpin). (C) *Cancer*. Water diffusion in the glioblastoma has been imaged and measured after the patient underwent a chemotherapy treatment. Areas within the tumor where the treatment has been efficient appear in green (return of water diffusion to baseline values), while areas in red highlight parts of the lesion where diffusion still remains low (courtesy B. Ross, University of Chicago). (D) *Functional brain imaging*. Areas in color represent parts of the brain (visual cortex) where water diffusion has slightly decreased during activation (visual presentation of a flickering checkerboard for 10 s).

## The birth of water diffusion MRI

Back in 1984 (when I was a radiology resident), a colleague came to me with a challenge: How could one differentiate liver tumors from angiomas? I had some fuzzy intuition that, perhaps, water molecular diffusion measurements would result in lower values in solid tumors because of steric hindrance to water molecular movement compared to flowing blood. Based on the pioneering work of physicists such as Stejskal and Tanner in the 1960s, I knew that specific encoding of the diffusion process could be achieved using magnetic field gradients, but the problem lied in the integration of such gradients with those used in the MRI scanner to generate images. The problem was not trivial and indeed many colleagues at that time thought that diffusion MRI was not feasible. The idea was to *localize* the diffusion measurements, that is, to map water diffusion coefficients in tissues; this had never been done before, especially *in vivo*. As a junior student in physics and medicine, I was very excited about this potential, and in a matter of weeks, diffusion MRI as we know it was conceived and implemented. My first diffusion images were obtained on a 0.5-tesla MRI scanner from the late CGR (Companie Générale de Radiologie, Buc, France). The first trials on liver were very disappointing, as the images were hampered by huge motion artifacts from respiration. This was due to the fact that the hardware to generate the magnetic gradients was sub-powered and also that the imaging methods at the time were slow and consequently very sensitive to patient motion. I thus decided to switch to the brain, as this was my original background and I must confess that I started scanning my own before actually moving to patients! It worked beautifully and that move established the great neurological potential for diffusion MRI; the rest, as the saying goes, is history.

The world's first diffusion images of the brain were made public in August 1985 at the Society of Magnetic Resonance in Medicine (SMRM) meeting in London. My first diffusion MRI paper appeared in 1985 in the journal of the French Academy of Sciences (Le Bihan & Breton, 1985). This paper described all the necessary ingredients to successfully perform diffusion MRI. The paper did not receive much attention, possibly because it was written in French. My next paper in *Radiology* (Le Bihan *et al*, 1986) was much better received, with more than 2000 citations up to now (3^rd^ most cited paper of all times for this journal). However, because of the sensitivity of diffusion MRI to motion artifacts, many colleagues remained sceptical at the time. It was discouraging, but I kept pressing on and it paid off, as diffusion MRI progressively gained momentum. Ironically, some of my early detractors started working full time on diffusion MRI. At the same time, it was becoming clear that the results of diffusion measurements with MRI in tissues would largely differ from those obtained for water in a glass where water diffuses freely, and I therefore introduced the *apparent diffusion coefficient* (ADC) concept to describe diffusion MRI findings (Le Bihan *et al*, 1986).

## Acute brain ischemia

Shortly thereafter, Michael Moseley at UCSF made an unexpected but crucial discovery in an acute cat brain ischemia model (Moseley *et al*, 1990): Water diffusion dropped significantly (30–50%) during the very early phase of acute brain ischemia. This finding tremendously boosted diffusion MRI, then still essentially a pure research tool, by attracting clinicians and convincing manufacturers to improve their systems. This move to the clinical field became possible after my encounter with Robert Turner at the National Institutes of Health (NIH) in Bethesda, where I had just moved. Dr Turner was an expert on EPI (Echo-Planar Imaging) MRI. With this technique, MRI images could be acquired as a snapshot in just a fraction of a second, virtually freezing patient motion: We were thus able to obtain the first “clean” diffusion images using EPI (Turner *et al*, 1990). This endeavor was not that simple, however, as we had to obtain gradient coils and power supplies with exceptional performances for the time, but we finally managed to obtain diffusion images in a matter of seconds (instead of minutes) and motion artifacts became history. The setup was also installed at Harvard Medical School in Boston where the first patients suffering from acute stroke (within 3–6 h of onset) were scanned with whole brain diffusion MRI in just a few seconds. The results immediately and directly confirmed Dr. Moseley's observations on cat brains: Water diffusion was found to be decreased in the infarcted areas, where dying brain cells undergo swelling through cytotoxic edema, clearly highlighting those areas as bright spots, while most of the time, standard MRI images (as well as CT scans) would not show any clear sign of abnormality (Warach *et al*, 1992). Around that time a drug company was developing an intravenous recombinant tissue plasminogen activator (rt-PA) drug aimed at thrombolytic therapy for acute stroke patients. Clearly, diffusion MRI could not have arrived at a better time, and this coincidental match became a milestone in the history of the management of acute stroke patients.

## The wirings of the brain

Michael Moseley's group had made another great discovery: In the diffusion images, contrast seemed to change according to the spatial direction of the diffusion measurement in white matter (spinal cord and brain). Water diffusion in white matter fibers was anisotropic, faster in the direction of the fibers and slower perpendicularly to them (and no one appeared aware of it until that time). With Philippe Douek, a French student working with me at the NIH, we suggested that this feature could be used to determine and map the orientation of white matter fibers in the brain, assuming the direction of the fibers was parallel to the direction with the fastest diffusion. The first attempt was very crude, with diffusion being measured along two directions only, but the concept of white matter fiber orientation color mapping was born and a proof of principle provided (Douek *et al*, 1991). Progress from those basic images to the gorgeous fiber tract 3D displays that now make up the covers of journals and anatomy textbooks, implied a big step, which was made possible by my encounter with Peter Basser in 1990. Peter was working at NIH on ionic fluxes in tissues. Peter quickly came up with the view that a better way to deal with anisotropic diffusion was to switch to a *tensor* formalism to properly determine the true direction of the highest diffusivity. The problem was to determine each of the terms of the diffusion tensor with diffusion MRI. After some brainstorming, Peter and I devised a solution in 1992, which we published and patented under the name of diffusion tensor imaging (DTI; Basser *et al*, 1994). Experiments were first conducted on vegetables with fibers, and soon after white matter fiber orientation could be obtained on a pixel-by-pixel basis within the whole brain *in vivo*, completely non-invasively and in just a few minutes. Algorithms were developed at the end of the 1990s to connect those pixels together, resulting in the world's first 3D representations of the fiber bundles (with very colorful representations of the “white” matter) within the human brain.

## Water, the forgotten biological molecule

Once back in France a few years later, my priority was to understand the basic mechanisms of water diffusion in biological tissues, which underlie what we visualize with diffusion MRI. This is a huge and certainly not a simple endeavor. We still cannot clearly explain why diffusion decreases so much in brain acute ischemia, how cell swelling leads to decreased diffusion, or precisely why diffusion anisotropy occurs in white matter. Some relevant and often sophisticated models have been proposed, but we always manage to find some piece of experimental evidence that questions those models. I have recently reviewed these inconsistencies and suggested that, beside mechanical or geometric constraints (such as cell membranes) hindering water diffusion, the physical structure of water networks in tissues, especially close to membranes, might play a role (Le Bihan & Johansen-Berg, 2012). The presence (or the amount) of structured water in cells is in itself a subject of great controversy among physicists and biologists, and we should be prepared for yet more exciting years of brainstorming and great workshops. I believe we have largely underestimated the importance of water in biology, from protein and membrane dynamics to cell physiology. This emerging research area should also greatly benefit from diffusion MRI.

## Imaging brain function

Another important possible application of diffusion MRI is the measurement of brain activity. Functional neuroimaging has become an essential means to study the brain and the mind. Thus far, positron emission tomography (PET) and current functional MRI (based on the Blood Oxygen Level Dependant or BOLD contrast mechanism) have relied on the principle that neuronal activation and blood flow are coupled through metabolism, and brain activation can be indirectly imaged through variations in local blood flow. With diffusion MRI, a new paradigm has emerged whereby we can look at brain activity through the observation of water molecular diffusion. In collaboration with my colleagues from Kyoto University, we have shown that water diffusion is indeed modulated by brain activity (Le Bihan *et al*, 2006). The diffusion signal response is characterized by a sharp peak, faster than the indirect hemodynamic response (increased in blood flow) observed with BOLD functional MRI imaging (Aso *et al*, 2009). The diffusion response persists after inhibition of neurovascular coupling (which suppresses the BOLD fMRI response) and shares the features of the underlying neuronal response (Tsurugizawa *et al*, 2013). This “diffusion fMRI” approach thus appears to be a paradigm shift in the way we visualize brain activity, and more directly linked to neuronal function, pointing out to changes in the neural tissue microstructure to which diffusion MRI is exquisitely sensitive. Such activation-driven structural events, for example cell swelling, have been reported in many instances in the literature. As cell swelling in tissues has been shown to result in a water diffusion decrease detectable with MRI, we have hypothesized that the slowdown in diffusion observed during neuronal activation could reflect cell swelling (probably at the dendrite and spine levels) occurring within the activated cortical ribbon. Based on this “electromechanical coupling,” the thought occurred to me that neural cells could perhaps be seen as piezoelectric sensors: Variations in cell shape should, in return, induce cell depolarization, potentially allowing a very fast, non-synaptic transmission mechanism within neural clusters of the cortical ribbon. Indeed, dynamic changes in neuronal structure (especially the dendritic spines) are now thought to play an important role in the functioning of such cell clusters, as envisioned by Crick (Crick, 1982) and even Ramon y Cajal (Ramon y Cajal, 1899): “The state of activity would correspond to the swelling and elongation of the [dendritic] spines, and the resting state (sleep or inactivity) to their retraction.” Diffusion MRI has the potential to address such questions and might allow us to further our understanding of the biophysical mechanisms associated with neuronal activation.

## Perspectives and conclusions

In summary, diffusion MRI has the potential to provide, non-invasively and *in vivo*, information on the cellular organization of the brain cortex, the connections between regions and the underlying activity. Still, the exact mechanisms governing water diffusion processes in tissues, notably in the brain, remain unclear. Future research should aim at gathering diffusion data at the individual neuron and neuron cluster scale to better understand water diffusion behavior in neural tissues. Indeed, a more profound understanding of such processes is necessary to develop the application of diffusion MRI further and to directly obtain information on tissue microstructure. Access to such micro- and mesoscopic scales will benefit from the unique ultra-high magnetic field MRI systems we have assembled in our institute dedicated to ultra-high field MRI (NeuroSpin, Saclay, France), in particular its preclinical 17.2-T MRI system and an experimental clinical 11.7-T MRI system (available from 2015), both unique in the world. Using magnetic resonance microscopy (MRM), we have shown that water diffusion measured inside isolated neuronal soma and in the region of cell bodies of the Aplysia buccal ganglia under exposure to ouabain, resulted in a water diffusion increase inside isolated neurons, but a decrease at the tissue level (Jelescu, 2014). Such opposite findings cannot be explained with current “mechanistic” tissue diffusion models. The scenario involving a layer of water molecules bound to the inflating cell membrane surface could conciliate this apparent discrepancy.

The potential of diffusion MRI to probe human brain connectivity has attracted worldwide interest and is now widely used in clinical practice. Recent results from the European FP7 CONNECT project (Assaf *et al*, 2013) and the Human Connectome Project (HCP, USA) have clearly underlined the enormous potential of this approach, yielding insight into how brain connections underlie function and opening up new lines of inquiry for human neuroscience and brain dysfunction in aging, mental health disorders, addiction and neurological disease (Le Bihan & Johansen-Berg, 2012). The increased spatial resolution expected with the NeuroSpin 11.7-T MRI scanner could allow for the detection of smaller white matter bundles. Of special interest are the short connections between adjacent cortical regions, or even within cortical regions. Similarly, there are also hints that diffusion imaging could become a very important tool to probe the functional architecture of the brain cortex. It has been long established that cortical cells are not organized along the brain cortex in a random, homogeneous way. Cells are rather well characterized (in terms of size, geometry, receptor type and density) and arranged in specific patterns, identified by Brodmann in 1908. The Brodmann areas are deemed to be associated with specific brain functions, and most functional neuroimaging studies have relied on the seminal classification of Brodmann to report the location of activated regions. With diffusion MRI, one may envision that cytoarchitectonic areas could be determined on an individual basis. Such specific cellular arrangements in space along the brain cortex might establish a “neural code,” a set of basic functions from which, once connected together on a timely manner, higher order functions could emerge.

While MRI is merely a means to visualize diffusion, molecular diffusion itself (of water or other molecules, metabolites, neurotransmitters) has a life of its own and remains a powerful, genuine multidisciplinary concept at our disposal to understand cell physiology and life. After all, all biological processes require molecules to interact, whether for DNA replication, RNA transcription, protein translation, protein and enzyme activity, cross-membrane transport, and so on. For molecules to interact, however, they must first meet: Diffusion appears to be the universal process through which this occurs. In a sense, diffusion rates set the speed limit for life, just as the speed of light sets the limit in the physical world. Indeed, diffusion MRI is just emerging from adolescence and has a great future.

## Conflict of interest

The author declares that he has no conflict of interest.
